# The Effects of Menstrual Cycle Phase on Gastrointestinal Responses to a Simulated Football Match

**DOI:** 10.1002/ejsc.70157

**Published:** 2026-03-13

**Authors:** S. J. Abbott, C. J. Parker, J. Hough, K. A. Hunter, M. A. Johnson, N. C. Williams

**Affiliations:** ^1^ Nottingham Trent University Sport Health and Performance Enhancement (SHAPE) Research Centre Nottingham UK

**Keywords:** football (soccer), gastrointestinal damage, gastrointestinal symptoms, gut barrier integrity, menstrual cycle

## Abstract

This study evaluated the effects of a simulated football match on gastrointestinal (GI) symptoms, damage and indirect markers of gut barrier integrity, and investigated whether GI responses are modulated by menstrual cycle (MC) phase. Twelve eumenorrheic females completed two 45‐min bouts of an intermittent treadmill protocol, replicating the activity profile of a football match, during Phase 1 (P1, days 1–5) and Phase 4 (P4, 6–8 days following a positive ovulation test) of their MC. Global GI discomfort was recorded every 15 min during exercise, and specific GI symptoms were assessed at rest, half‐time (HT), full‐time (FT) and 60 min post‐exercise (POST‐60). Blood samples were collected at rest, FT and POST‐60 to assess intestinal fatty‐acid binding protein (I‐FABP), lipopolysaccharide binding protein (LBP), soluble cluster of differentiation 14 (sCD14) and claudin‐3 (CLDN‐3). I‐FABP increased by 51% from rest to FT (*p* = 0.007), but there was no effect of exercise on LBP, CLDN‐3 or sCD14. Global GI discomfort was 65% greater in P1, than P4 (*p* = 0.006) and total GI symptom score was greater in P1 than P4 at rest (*p* = 0.011) and FT (*p* = 0.021). CLDN‐3 concentrations were greater in P1 than P4 at rest (*p* = 0.02) and POST‐60 (*p* = 0.03). There were no differences between MC phase for I‐FABP, LBP or sCD14. Participants experienced increased GI discomfort during P1 compared to P4 of the MC, at rest and during exercise. However, exercise‐induced GI symptoms and damage occurred at a similar rate in both MC phases.

## Highlights

- Football‐specific intermittent treadmill running induced significant gastrointestinal damage from rest to full‐time in both Phase 1 and Phase 4 of the menstrual cycle, indicating substantial exercise‐induced enterocyte injury, irrespective of menstrual cycle phase.
- Gastrointestinal symptoms were elevated in Phase 1, compared to Phase 4, of the menstrual cycle at rest, during and following a simulated football match, with significantly higher global gastrointestinal discomfort and total gastrointestinal symptom scores reported.
- The increased gastrointestinal symptom burden in Phase 1 was not associated with changes in gut barrier integrity. The mechanisms driving menstrual cycle phase‐related gastrointestinal symptoms therefore require further investigation.
- These findings highlight the importance of routine gastrointestinal symptom screening for female athletes, to enable early identification and targeted management of any menstrual cycle phase‐related changes in gastrointestinal symptoms, particularly around exercise.

## 1 Introduction

Exercise‐induced gastrointestinal (GI) symptoms, such as nausea, abdominal cramps, diarrhoea and vomiting, have been reported to affect 30%–90% of athletes and can have a detrimental effect on exercise performance (de Oliveira and Burini 2009). Recent evidence from applied and survey‐based studies suggests that the prevalence and severity of GI symptoms may be greater in female compared with male team sport athletes (Wilson et al. 2023; Chantler et al. 2024). Female team sport athletes reported higher GI symptom scores at rest, during training and in competition than males, and 37.5% of females reported that their performance had been negatively impacted by GI symptoms, compared to 13.9% of males (Wilson et al. 2023). Female rugby players have also reported greater frequency and severity of GI symptoms than their male counterparts at rest and around training and competition (Chantler et al. 2024).

Although the cause(s) of exercise‐induced GI symptoms is unknown, several mechanisms have been proposed: splanchnic hypoperfusion and subsequent intestinal ischaemia causing GI damage and increased permeability (van Wijck et al. 2011); sympathetic activation and impaired GI function (Leiper 2015; Costa et al. 2017); intestinal injury caused by gastric jostling during running exercise (de Oliveira et al. 2014) and reduced epithelial barrier integrity (K. A. Smith et al. 2021). Damage to the intestinal barrier during exercise can result in increased leakage of pathogenic gram‐negative bacteria containing lipopolysaccharides into the circulation, triggering an immune response and the subsequent production of pro‐ and anti‐inflammatory cytokines, which may contribute to GI symptoms (Lambert 2009). In addition, exercise‐induced activation of the sympathetic nervous system and associated neuroendocrine responses may reduce GI motility, transit, and overall functional capacity, contributing to the development of GI symptoms (Leiper 2015; Costa et al. 2017).

Because of difficulties in directly assessing GI barrier integrity and microbial translocation, surrogate blood biomarkers are often used (Ogden et al. 2020). Intestinal fatty‐acid binding protein (I‐FABP) is expressed in the intestinal epithelial cells and is released extracellularly following enterocyte injury (Pelsers et al. 2003). Consequently, I‐FABP is commonly used as a sensitive marker of acute GI damage and has been shown to increase in response to a range of exercise modes and intensities (Chantler et al. 2021). High circulating levels of lipopolysaccharide binding protein (LBP) and soluble cluster of differentiation 14 (sCD14) are often used as indirect surrogate markers of gut barrier integrity because they respond to lipopolysaccharides (Wright et al. 1990) however they remain non‐specific and indirect indicators because they can also increase for other reasons unrelated to GI integrity or LPS translocation. Claudin‐3 (CLDN‐3) is a tight junction protein mainly found in the colon and duodenum. The presence of CLDN‐3 in the circulation indicates the dislodging of the tight junction proteins and has, therefore, also been proposed as an indirect intestinal permeability marker (Yeh et al. 2013). Whereas there is some uncertainty about the relevance of these markers to GI symptoms, studies have shown a positive correlation between exercise‐induced GI symptoms, and increases in I‐FABP, LBP, CLDN‐3 (McKenna, Fennel, et al. 2022) and sCD14 (Pugh et al. 2019). There has been a predominance for exercise and GI research to focus on the endurance athlete; however, high intensity intermittent running has also been shown to increase GI damage and permeability (McKenna, Houck, et al. 2022). Research into the effect of team sport exercise, which is characterised by high intensity bursts of activity, on GI symptoms, damage and permeability is lacking, particularly in female athletes.

The high prevalence of GI symptoms in female athletes might be influenced by the menstrual cycle (MC). The MC can be categorised into four distinct phases, based on ovarian hormone profiles: Phase 1 (P1), lowest concentrations of oestrogen and progesterone; Phase 2, highest oestrogen and low progesterone concentrations; Phase 3, medium oestrogen and low progesterone concentrations and Phase 4 (P4), high oestrogen and highest progesterone concentrations (Elliott‐Sale et al. 2021). At rest, females experience a higher incidence and severity of GI symptoms during P1 of their MC compared to all other MC phases (Pugh et al. 2022), with symptoms such as bloating, diarrhoea, and nausea commonly reported (Bruinvels et al. 2021; McNulty et al. 2023). Symptoms of diarrhoea and abdominal pain, in addition to other MC symptoms, may contribute to perceived reductions in exercise performance and an increased likelihood of missing or adapting training or competition (Armour et al. 2020; Bruinvels et al. 2021; Parker et al. 2022). In contrast to other elite female sports where 47%–50% of players report hormonal contraceptive use (Martin et al. 2018; Larsen et al. 2020), Parker et al. (2022) found that 72% of Women's Super League football players do not use hormonal contraceptives. Therefore, research into the effect of the MC and associated symptomology in non‐hormonal contraceptive users is particularly important in female football players.

Dual sugar carbohydrate ratio tests, such as the lactulose/rhamnose (L/R) and lactulose/mannitol (L/M) tests, are commonly used to assess GI barrier permeability (Camilleri et al. 2010). There is limited evidence investigating the effect of MC phase on GI permeability in humans. Flood et al. (2022) demonstrated that, at rest, there were no differences in L/R urinary excretion ratio between phases 1, 2 and 4. Similarly other studies demonstrate no difference in GI permeability, as assessed by L/R and L/M ratio, between MC phases at rest (Torella et al. 2007; Lambert 2009). These studies were all completed at rest in healthy women, where baseline levels of GI damage and permeability are likely low. It is well established that exercise stress can lead to increased GI permeability, particularly during higher duration or intensity exercise (de Oliveira and Burini 2009). However, whether exercise has different effects on gut barrier integrity and damage during different phases of the MC is unknown. Therefore, the aim of this study was to assess the effects of a simulated football match on GI symptoms, damage and indirect markers of gut barrier integrity, and determine whether GI responses are modulated by MC phase.

## 2 Materials & Methods

### 2.1 Participants

Twelve healthy females (age = 26.8 ± 4.4 years; height = 166.4 ± 7.2 cm; body mass = 65.9 ± 10.2 kg; $\dot{V}$O_2max_ = 47.8 ± 6.7 mL/kg/min) took part in this study. Eighteen participants initially volunteered to participate; however, six participants were withdrawn due to MC irregularity and/or injury. Inclusion criteria required participants to be eumenorrheic (Elliott‐Sale et al. 2021) with menses occurring at regular intervals of 21–35 days. The average cycle length was 29 ± 2 days, and a positive ovulation test (One‐step, Hangzhou Alltest Biotech Co. Ltd., China) was recorded on day 14 ± 1. Participant blood samples were analysed to retrospectively confirm correct hormone profiles. Participants had not used hormonal contraceptives in the 6 months prior to the study and reported being free from other MC‐related irregularities or conditions. All participants were classified as trained (83%) or highly trained (17%) (McKay et al. 2022) and were recruited from football (*n* = 5), rugby union (*n* = 5) and hockey (*n* = 2). Participants were informed of the risks associated with the study and provided written, informed, consent prior to study involvement. All procedures were performed in accordance with the Declaration of Helsinki, except for registration in a database, and were approved by the Nottingham Trent University Human Invasive Ethics Committee (REF: 702).

### 2.2 Study Design

Participants attended the laboratory on four occasions. During the first laboratory visit, participants completed exercise tests on a motorised treadmill (HP Cosmos, Germany) set at a 1% gradient. Participants completed a speed lactate test, consisting of 3‐min stages, starting at 8 km/h and increasing by 1 km/h at the beginning of each stage. At baseline, and immediately following each stage, a fingertip capillary blood sample was collected into a haematocrit capillary tube for the determination of blood lactate concentration (Biosen C‐line, EKF diagnostics, UK). The test continued until the maximal lactate steady state (MLSS) velocity was achieved, which was defined as the fastest speed with less than a 1 mmol/L increase in blood lactate concentration above the preceding value (Åstrand et al. 2003). Following 10 min of static rest, the maximal oxygen uptake ($\dot{V}$O_2max_) of each participant was then determined via an incremental test, starting at their MLSS velocity and increasing by 1 km/h every minute until task failure. Expired gas was measured breath‐by‐breath using a metabolic cart (Vyntus CPX, Vyaire Medical Inc, UK) and values were averaged over 10 s. $\dot{V}$O_2max_ was taken as the highest $\dot{V}$O_2_ over the 10‐s averages. The following endpoint criteria were used to determine whether $\dot{V}$O_2max_ was achieved: plateau in $\dot{V}$O_2_ profile; heart rate within 10 beats/min of age predicted maximum and respiratory exchange ratio > 1.1.

**FIGURE 1:**
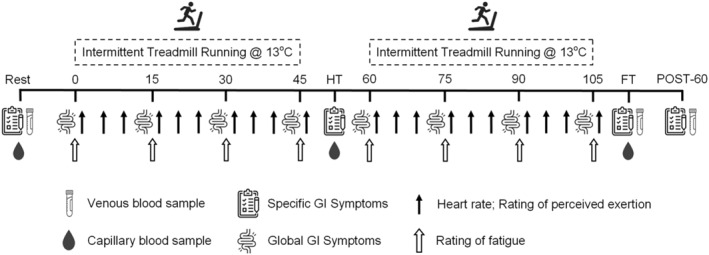
Experimental trial protocol. FT, full‐time; GI, gastrointestinal; HT, half‐time; POST‐60, 60 min post‐exercise.

During the second laboratory visit, participants completed a familiarisation trial, which involved the same protocol as in the experimental trials (Figure 1), up until the half‐time (HT) period. The two experimental trials occurred during P1 (indicated by the onset of bleeding until day 5) and P4 (6–8 days following a positive ovulation test) of the participants' MC, as defined by Elliott‐Sale et al. (2021). A crossover order‐balanced study design was used. Six participants completed both experimental trials during the same MC (P1 trial followed by P4 trial) and six participants completed the experimental trials in two consecutive MCs (P4 trial followed by P1 trial).

### 2.3 Experimental Trials

Participants arrived at the laboratory at the same time of day for each trial. Participants were instructed to arrive at least 2 hours post‐prandial, to have avoided strenuous exercise and alcohol during the previous 24 h, and caffeine in the previous 12 h. Participants completed a food diary in the 24 h prior to their first experimental trial, which was then replicated prior to their next experimental trial. During the first experimental trial, participants consumed water *ad libitum*, with intake ranging from 230–850 mL, which was then matched during their next experimental trial.

Both experimental trials were performed in an environmental chamber (TISS series 201003–1, TIS services, UK) set at 13°C and 60% Rh, based on the average UK temperature across the 2021/22 football season (Met Office, Hadley Centre, UK), with a fan used to simulate outdoor airflow. Each experimental trial consisted of two 45‐min bouts of football‐specific intermittent treadmill running (Figure 1), separated by a 15‐min rest period to simulate HT, where participants were seated outside of the environmental chamber. Venous blood samples were collected at rest, full‐time (FT) and 60 min post‐exercise (POST‐60) and capillary blood samples were collected at rest, HT and FT. Participants completed a specific GI symptom questionnaire (Gaskell et al. 2019) at rest, HT, FT and POST‐60. Heart rate (Polar H10, Finland) and rating of perceived exertion (RPE) (Borg 1982) were recorded every 5 min throughout exercise. Global GI discomfort (Nieman et al. 2006) and subjective rating of fatigue (Micklewright et al. 2017) were recorded every 15 min.

### 2.4 Football‐Specific Intermittent Treadmill Protocol

A football‐specific intermittent treadmill protocol was used to replicate the physical demands of a football match. The protocol comprised of seven different treadmill speeds (Table 1), which were interspersed throughout the 45‐min protocol to simulate the activity pattern of a football match and were completed at a 1% gradient to simulate outdoor running. The protocol was adapted from Greig et al. (2006) to include individualised treadmill speeds, which were determined by participants' velocity at $\dot{V}$O_2max_ and MLSS. The average distance covered by participants during the two 45‐min bouts of the football‐specific intermittent treadmill protocol was 10461 ± 716 m.

**TABLE 1:** Overview of the activities, speeds and durations of the 45‐min football‐specific intermittent treadmill protocol.

Activity	Speed (*km/h*)	Number of activities	Mean duration (*s*)
Standing	0	60	7.8
Walking	4	155	6.7
Jogging	Velocity before MLSS	125	3.5
Low speed	85% v$\dot{V}$O_2max_	138	3.5
Moderate speed	100% v$\dot{V}$O_2max_	60	2.5
High speed	125% v$\dot{V}$O_2max_	27	4.0
Sprint	145% v$\dot{V}$O_2max_	9	2.0

Abbreviations: MLSS, maximal lactate steady state; v$\dot{V}$O_2max_, velocity at $\dot{V}$O_2max_.

### 2.5 Assessment of GI Symptoms

#### 2.5.1 Global GI Discomfort

Global GI discomfort was assessed every 15 min throughout exercise on a 10‐point scale (Nieman et al. 2006) ranging from 0 (no discomfort) to 9 (worst it has ever been). Participants were asked to consider any feelings of GI discomfort such as nausea and flatulence.

#### 2.5.2 Specific GI Symptom Questionnaire

Participants rated the severity of specific GI symptoms on a 10‐point scale, with 0 indicating an absence of symptoms, 1–4 indicating mild symptoms, 5–8 indicating severe symptoms, and 9–10 indicating extremely severe symptoms (Gaskell et al. 2019). The presence of either regurgitation, projectile vomiting or defaecation were rated as either 0 (not present) or 10 (extremely severe). All symptom scores were summed to generate a total GI symptom score at each time‐point, and symptoms associated with the upper and lower GI tract were separated to determine regional symptom scores. The maximum possible score was 180 for total GI symptoms, 70 for upper GI tract symptoms and 80 for lower GI tract symptoms.

### 2.6 Blood Sampling and Analysis

Capillary blood samples were collected via fingerprick method for the determination of blood lactate concentration (Biosen c‐line, EKF Diagnostics, Germany). Venous blood samples were collected via the antecubital vein using a 23‐gauge butterfly needle and lithium heparin and K_2_EDTA containing vacutainers. Once drawn, samples were placed on ice, prior to centrifugation (10,000–15,000*g*, 10–15 min, 4°C), as per manufacturer's instructions. Plasma samples were subsequently frozen at −80°C until analysis via enzyme‐linked immunosorbent assay (ELISA). GI damage was assessed by measuring I‐FABP (Hycult Biotechnology, Uden, the Netherlands); and indirect markers of gut barrier integrity were assessed by measuring LBP (Hycult Biotechnology, Uden, the Netherlands), sCD14 (R&D systems, Minneapolis, USA) and CLDN‐3 (Elabscience, Houston, USA). A resting venous blood sample was collected to confirm MC phases, through analysis of 17‐β oestradiol and progesterone (Cayman Chemical, Michigan, USA). P1 was confirmed by low levels of 17‐β oestradiol and progesterone, and P4 was confirmed by a greater 17‐β oestradiol concentration than in P1, and a progesterone concentration > 6.4 ng/mL (Elliott‐Sale et al. 2021). Centrifugation and ELISA kit procedures were performed according to the manufacturer's instructions. The intraassay coefficient of variations were 6.7% for I‐FABP; 8.3% for LBP; 6.4% for sCD14; 7% for CLDN‐3; 8.5% for 17‐β oestradiol and 4.8% for progesterone.

### 2.7 Statistical Analysis

Statistical analyses were performed using the statistical package for social sciences (IBM SPSS version 29, IBM Corp, New York, USA). Normality of distribution of dependent variables was assessed using the Shapiro–Wilk test. The level of significance was set at *α* = 0.05.

As data relating to subjective ratings of GI symptoms were not normally distributed a non‐parametric approach was utilised. Data analysed using non‐parametric tests are presented as median (interquartile range, IQR), unless otherwise stated. Effect sizes were calculated as the *Z* statistic divided by the square root of N, where ≥ 0.1 indicated a small effect, ≥ 0.3 a moderate effect and ≥ 0.5 a large effect (Cohen 1988). The area under the curve (AUC) of global GI discomfort during exercise was determined using the trapezoidal rule. Wilcoxon signed‐rank tests were conducted to compare the AUC of global GI symptoms between MC phases as well as to evaluate the absolute change in global GI discomfort from baseline to FT. Total, lower and upper GI tract symptom scores were analysed using Friedman's test to assess main effects of time and Wilcoxon signed‐rank tests to assess main effects of MC phase and for post hoc analysis.

Data evaluated using parametric tests are presented as mean ± standard deviation, unless otherwise stated. Effect sizes were determined using Cohen's d (1988), where 0.20–0.49 indicated a small effect, 0.50–0.79 a moderate effect, and ≥ 0.80 a large effect. Between MC phase comparisons for 17‐β oestradiol and progesterone concentration were conducted using paired sample *T*‐tests. I‐FABP, CLDN‐3, LBP and sCD14 concentrations were analysed using a two‐way (MC phase x time) repeated measures analysis of variance (ANOVA). All significant main effects and interactions were assessed further with pairwise comparisons using Bonferroni corrections. Between MC phase comparisons for mean heart rate, RPE, rating of fatigue and blood lactate concentration were also conducted using paired sample *T*‐tests.

## 3 Results

### 3.1 Global GI Discomfort

**FIGURE 2:**
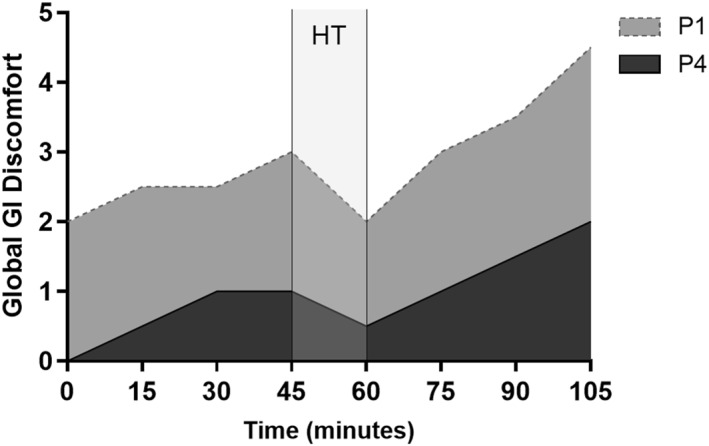
Rating of global GI discomfort during 2 × 45‐min bouts of a football‐specific intermittent treadmill protocol separated by a 15‐min rest period (*N* = 12). A Wilcoxon signed‐rank test revealed differences in global GI discomfort between menstrual phases (*p* = 0.006). GI, gastrointestinal; HT, half‐time; P1, phase 1; P4, phase 4.

Global GI discomfort increased from 0 to 105 min by 2.5 in P1 (*p* = 0.003, *r* = 0.60) and by 2.0 in P4 (*p* = 0.008, *r* = 0.55). The AUC of global GI discomfort experienced during the football‐specific intermittent treadmill protocol (Figure 2) was greater in P1 (22.8 [9.5–24.6]), than in P4 (13.8 [2.9–21.5]) (*p* = 0.006) and a large effect size was observed (*r* = 0.50). The change in global GI discomfort from baseline was not different between MC phases (*p* = 0.857).

### 3.2 Specific GI Symptoms

#### 3.2.1 Total GI Symptom Score

**FIGURE 3:**
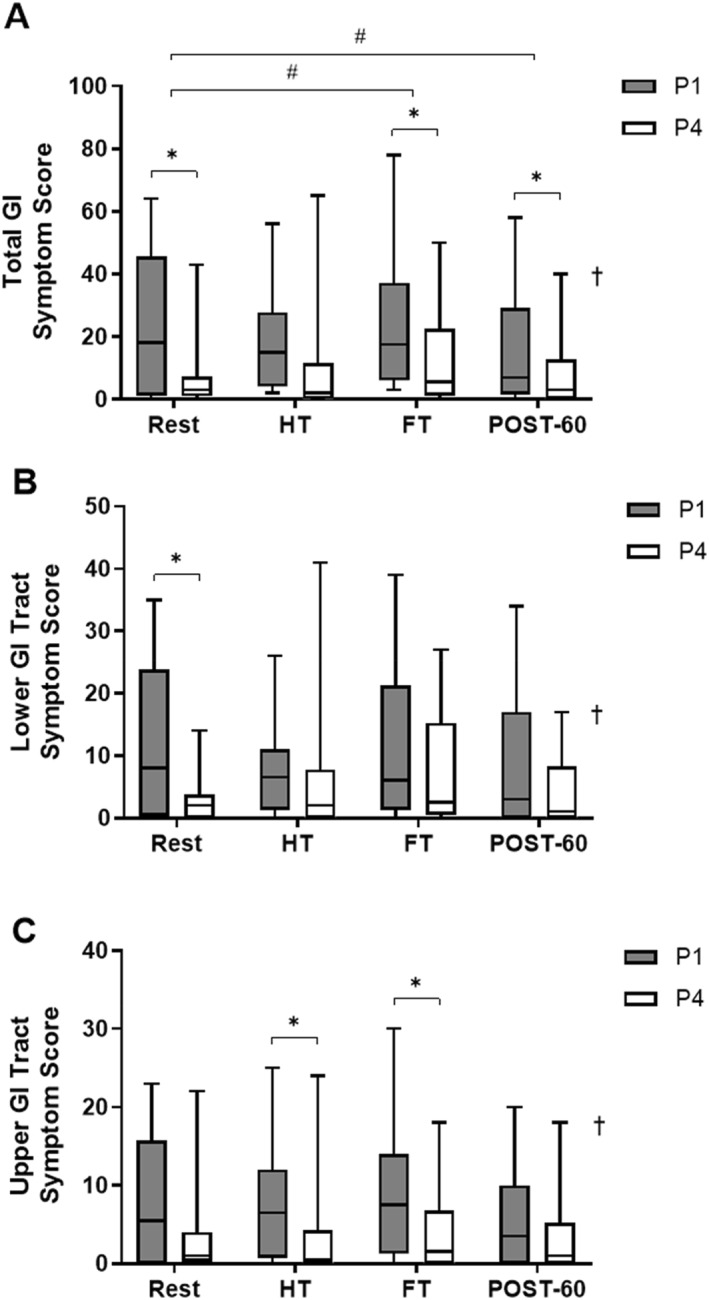
Rating of (A) total, (B) lower, and (C) upper GI tract symptom score in response to a football‐specific intermittent treadmill protocol (*N* = 12). Data is presented as median (IQR): † main effect of MC phase (*p* < 0.05), # significant difference versus rest (*p* < 0.05), ^✱^ significant difference between MC phases at the corresponding time‐point (*p* < 0.05). GI, gastrointestinal; HT, half‐time; FT, full‐time; POST‐60, 60 min post‐exercise; P1, phase 1; P4, phase 4.

There was a main effect of time (χ^2^(3) = 18.342, *p* < 0.001) on total GI symptom score (Figure 3A). There was no change in total GI symptom score from rest to HT (*p* = 0.637). Total GI symptom score was higher at FT, compared to rest (*p* = 0.039) and was lower at POST‐60 than rest (*p* = 0.038). There was also a main effect of MC phase (*Z* = −4.401, *p* < 0.001), with total GI symptom score greater in P1, compared to P4, at rest (*p* = 0.011, *r* = 0.52) and FT (*p* = 0.021, *r* = 0.47).

#### 3.2.2 Lower GI Tract Symptom Score

For lower GI tract symptom score (Figure 3B), there was a main effect of time (χ^2^(3) = 12.995, *p* = 0.005). However, lower GI tract symptom score was unchanged from rest at HT (*p* = 0.367), FT (*p* = 0.265), and POST‐60 (*p* = 0.208). There was also a main effect of MC phase (*Z* = −3.030, *p* < 0.002) and lower GI tract symptom score at rest was higher in P1 compared to P4 (*p* = 0.016, *r* = 0.49).

#### 3.2.3 Upper GI Tract Symptom Score

There was a main effect of time (χ^2^(3) = 8.466, *p* = 0.037) on upper GI tract symptom score (Figure 3C). However, upper GI tract symptom score was unchanged from rest at HT (*p* = 0.531), FT (*p* = 0.199) and POST‐60 (*p* = 0.106). There was also a main effect of MC phase (*Z* = −4.234, *p* < 0.001). There were no differences in upper GI tract symptom score between MC phases at rest (*p* = 0.073, *r* = 0.37), but upper GI tract symptom score was higher in P1, compared to P4, at HT (*p* = 0.011, *r* = 0.52) and FT (*p* = 0.016, *r* = 0.49).

#### 3.2.4 GI Symptom Incidence

**FIGURE 4:**
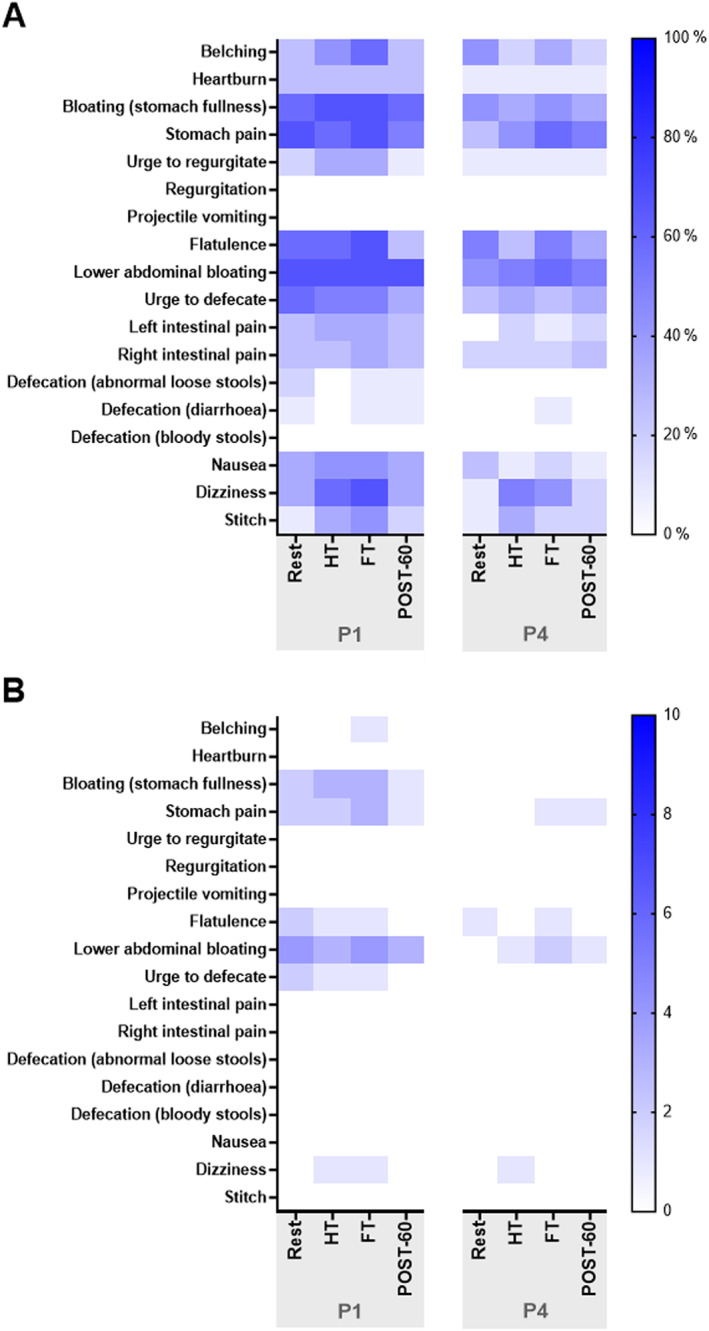
(A) Percentage incidence and (B) median severity of gastrointestinal symptoms reported in response to a football‐specific intermittent treadmill protocol (*N* = 12). HT, half‐time; FT, full‐time; POST‐60, 60 min post‐exercise; P1, phase 1; P4, phase 4.

The most commonly reported GI symptoms across both P1 and P4 were bloating, flatulence, lower abdominal bloating and stomach pain (Figure 4A) and participants reported the greatest incidence of GI symptoms at FT. In P1, the most commonly reported GI symptoms were bloating, flatulence, lower abdominal bloating and stomach pain, experienced by eight (67%) participants at FT. In P4, stomach pain and lower abdominal bloating were the highest reported symptoms, reported by seven (58%) participants. Whereas similar GI symptoms were reported in both MC phases, they were found to have the greatest incidence (Figure 4A) and severity (Figure 4B) during P1.

### 3.3 Sex Hormones

**FIGURE 5:**
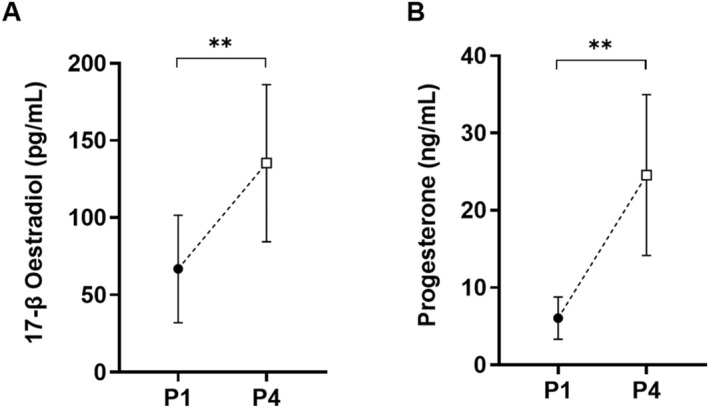
Resting (A) 17‐β oestradiol and (B) progesterone concentrations in phase 1 (P1) and phase 4 (P4) of the menstrual cycle (MC) (*N* = 12). Mean ± SD: ^✱✱^ significant difference between MC phases (*p* < 0.01).

Concentrations of 17‐β oestradiol and progesterone were greater in P4 compared to P1 (*p* < 0.001) (Figure 5).

### 3.4 GI Damage

**FIGURE 6:**
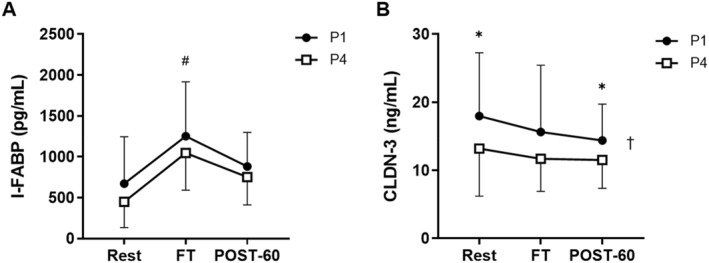
Concentrations of (A) I‐FABP and (B) CLDN‐3 in response to a football‐specific intermittent treadmill protocol (*N* = 12). Mean ± SD: † main effect of menstrual cycle (MC) phase (*p* < 0.05), # significant difference versus rest (*p* < 0.05), ^✱^ significant difference between MC phases at the corresponding time‐point (*p* < 0.05). I‐FABP, intestinal fatty acid binding protein; CLDN‐3, claudin‐3; FT, full‐time; POST‐60, 60 min post‐exercise; P1, phase 1; P4, phase 4.

There was a main effect of time on I‐FABP concentration (*p* < 0.001) (Figure 6A). I‐FABP concentration increased from rest to FT (*p* = 0.007, *d* = 1.29) but was not different from rest at POST‐60 (*p* = 0.218, *d* = 0.56). There was no main effect of MC phase (*p* = 0.096) on I‐FABP concentration and no interaction effect (*p* = 0.847).

### 3.5 Indirect Markers of Gut Barrier Integrity

For CLDN‐3 concentration (Figure 6B), there was a main effect of time (*p* = 0.046) but, compared to the rest, there were no differences in CLDN‐3 concentration at FT (*p* = 0.077) or POST‐60 (*p* = 0.146). There was also a main effect of MC phase (*p* = 0.016), but no interaction effect (*p* = 0.611). CLDN‐3 concentrations were greater in P1 than P4 at rest (*p* = 0.029; *d* = 0.61) and POST‐60 (*p* = 0.034; *d* = 0.60). At FT, there was no difference in CLDN‐3 concentration between MC phases (*p* = 0.077), but there was a medium effect size (*d* = 0.51).

For LBP concentration, there were no main effects of time (*p* = 0.080) or MC phase (*p* = 0.687), and no interaction effect (*p* = 0.779). Similarly, for sCD14 concentration, there was no main effects of time (*p* = 0.241) or MC phase (*p* = 0.787) and no interaction effect (*p* = 0.932).

### 3.6 Physiological Measures

Resting heart rate was higher in P4 (89 ± 13 bpm) compared to P1 (83 ± 13 bpm) (*p* = 0.033; *d* = 0.59). Average heart rate throughout exercise was greater during P4 (148 ± 12 bpm) compared to P1 (142 ± 12 bpm) (*p* = 0.003; *d* = 1.0). During exercise, there were no differences between MC phases for mean RPE (P1: 13 ± 2 vs. P4: 13 ± 1) (*p* = 0.269), or rating of fatigue (P1: 4 ± 2 vs. P4: 3 ± 1) (*p* = 0.127). There were also no differences between MC phases for blood lactate concentration at rest (P1: 1.14 ± 0.28 mmol/L vs. P4: 1.24 ± 0.31 mmol/L) (*p* = 0.317), HT (P1: 2.04 ± 0.78 mmol/L vs. P4: 2.04 ± 0.66 mmol/L) (*p* = 0.990) or FT (P1: 2.14 ± 0.76 mmol/L vs. P4: 2.11 ± 0.82 mmol/L) (*p* = 0.931).

## 4 Discussion

The aims of this study were to assess the effects of a simulated football match on GI symptoms, damage and indirect markers of gut barrier integrity and determine whether these GI responses are modulated by MC phase. In response to exercise, participants reported increased levels of GI discomfort, with the greatest incidence and severity of GI symptoms reported immediately post‐exercise. Bloating, flatulence, lower abdominal bloating and stomach pain were the most commonly reported GI symptoms across both MC phases. Following the simulated football match, there was an increase in the gut damage marker, I‐FABP, compared to rest but there were no changes in indirect markers of gut barrier integrity (CLDN‐3, LBP and sCD14) in response to exercise. The primary finding of this study was that GI symptoms experienced pre‐, during and post‐a simulated football match were greater during P1 compared to P4 of the MC. We did not observe any effect of MC phase on I‐FABP, LBP or sCD14 concentrations, but CLDN‐3 concentrations were greater in P1 than P4 at both rest and POST‐60.

In response to the simulated football protocol, there was a large 51% increase in I‐FABP in both P1 and P4, which suggests that football‐type exercise is sufficient to induce GI damage in female athletes, even in temperate conditions (13°C) typical of the UK football season. This increase is in accordance with a recent meta‐analysis which identified increases in I‐FABP following running, cycling and resistance training exercise (Chantler et al. 2021). We did not observe any changes in CLDN‐3, LBP or sCD14 in response to the simulated football protocol, suggesting that there were no exercise‐induced changes in indirect surrogate markers of gut barrier integrity in the present study. This contrasts with Yeh et al. (2013) who found CLDN‐3 concentration in males to increase following 60 min of running at 70% $\dot{V}$O_2_max, in both hot (30°C) and mild (22°C) conditions. McKenna, Fennel, et al. (2022) also observed an increase in CLDN‐3 and LBP post‐exercise in males, in response to 60 min of cycling at 65% $\dot{V}$O_2_max in hypoxia, but not in normoxia. Following 60 min of high‐intensity interval exercise in the heat (40°C), McKenna, Fennel, et al. (2022), found no changes in CLDN‐3 concentration, however, they did observe an increase in LBP in response to exercise in males. These studies do, however, highlight some inconsistencies within the literature, regarding exercise‐induced changes in circulating CLDN‐3. There are a limited number of studies which have assessed the impact of exercise on sCD14, but sCD14 has been shown to increase from pre‐to post‐race, following marathon running in a mixed cohort (Pugh et al. 2019). This may indicate that, at lower ambient temperatures (13°C), football‐type exercise does not cause an acute increase in indirect markers of gut barrier integrity in female athletes, and that exercise‐induced GI permeability is more likely to occur following longer duration exercise, or exercise in extreme environments (Costa et al. 2017; Chantler et al. 2021).

We observed a large effect of MC phase on total GI symptom score at rest, with participants experiencing greater levels of GI symptoms in P1. Similarly, lower GI tract symptom score was elevated in P1 compared to P4 at rest. These results are consistent with previous research reporting higher resting GI symptoms during P1 of the MC (Bruinvels et al. 2021; McNulty et al. 2023). At rest, there was no difference in upper GI tract symptoms between MC phases, suggesting that resting MC‐related GI symptoms predominantly impact the lower GI tract. Whereas exercise‐induced changes in global GI discomfort were similar in P1 and P4, the higher global GI discomfort at rest in P1 resulted in a 65% greater burden of exercise‐induced GI discomfort, compared to P4. Participants also reported greater total and upper GI tract symptom scores at FT in P1 than in P4. The current study demonstrates that the elevated levels of GI discomfort experienced at rest in P1 translates to continued worsening of GI symptoms when exercising.

Sex hormones may play a modulatory role in intestinal barrier function through the activation of oestrogen receptors (ERα and β), which are expressed in intestinal epithelial cells throughout the GI tract (Nie et al. 2018). In murine (Braniste et al. 2009), organoid and cell line (van der Giessen et al. 2019) models, oestrogen has been proposed to improve epithelial barrier function via ERβ‐mediated up‐regulation of tight junction proteins. The claudin family of tight junction proteins are integral in the regulation of the semi‐permeable membrane between epithelial cells (Groschwitz and Hogan 2009). In the present study, circulating levels of CLDN‐3 were 33% greater in P1, compared to P4, which may indicate greater dislodging of tight junction proteins during P1 of the MC. Similarly, van der Giessen et al. (2019) showed improved epithelial barrier strength and an up‐regulation of CLDN‐1 and ‐2 tight junction protein expression in cell line and organoid models, in response to treatment with oestrogen and progesterone. Li et al. (2016) also observed a weakened intestinal barrier integrity and reduced transcript levels of CLDN‐2, ‐3 and ‐15, following pharmacologically induced sex steroid depletion in female mice. Taken together, these studies could indicate a weakening of the intestinal barrier during low oestrogen periods, for example during P1 of the MC, but this requires further empirical evidence.

However, without performing an intestinal biopsy, it is not possible to directly assess intestinal tight junction breakdown. As a relatively new biomarker, the physiological relevance of elevated CLDN‐3 remains poorly understood (Ogden et al. 2020), which presents a limitation in the interpretation of these data. Although CLDN‐3 is one of the most highly expressed claudin proteins in the gut and has been proposed as an indirect surrogate marker of gut barrier integrity (Yeh et al. 2013), it is also expressed in a variety of other tissues (Günzel and Yu 2013). Because of the nature of assessing this marker through venous blood samples, it is not possible to determine the site of origin of circulating CLDN‐3. Elevated levels of circulating CLDN‐3 cannot, therefore, provide a specific indication of GI permeability, but may instead reflect greater systemic tight junction dysregulation. Importantly, despite greater CLDN‐3 concentrations in P1, compared to P4, there were no differences in levels of circulating sCD14, or LBP between MC phases at any time‐point. Similarly, Flood et al. (2022) did not observe any significant differences in GI permeability at rest across the MC, despite observing an elevation in GI symptoms in P1, compared to phases 2 and 4. This suggests that the differences in GI symptoms experienced between MC phases are not driven by changes in intestinal barrier integrity.

The cause of elevated levels of resting GI symptoms in P1 is likely multi‐factorial. For example, fluctuations in oestrogen and progesterone throughout the MC may affect the occurrence of GI symptoms due to changes in visceral sensitivity and GI motility (Meleine and Matricon 2014). Additionally, during P1, there is a rise in prostaglandins, in particular PGF_2α_, which stimulate uterine contractions to facilitate menstrual shedding (Judkins et al. 2020). Because of the proximity between the uterus and GI tract, this may also increase smooth muscle contractions in the intestines, contributing to cramping and diarrhoea‐type symptoms. Oestrogen has also been reported to have anti‐inflammatory and antioxidant properties (Whitcomb et al. 2014), and changes in inflammatory responses across the MC may also impact exercise associated GI symptoms (Lambert 2009).

The increased number of GI symptoms reported by participants both at rest and during exercise in P1 may have important practical implications for female athletes. Although we did not directly assess the impact of GI symptoms on exercise performance in the present study, female team sport athletes have previously reported GI symptoms to negatively affect their sporting performance (Wilson et al. 2023). In a recent study by E. S. Smith et al. (2025), the number of at least ‘moderate’ severity GI symptoms reported by female athletes before a virtual time‐trial cycling race were positively correlated with increased (i.e., slower) race time. The number of perceived MC symptoms also correlated with reduced race performance, highlighting the importance of GI and MC symptom management in optimising exercise performance. Whereas this suggests an impact of MC‐related GI symptoms on endurance performance–an important component of football–these symptoms could affect other aspects of performance in football, such as decision‐making and execution of technical skills. Increased GI symptoms experienced in P1 may also have an indirect influence on performance, through potential impacts on nutritional behaviours pre‐, during or post‐exercise (Parnell et al. 2020). Interviews with elite female football players (Read et al. 2022), revealed that 47% of players experienced a decrease in appetite during P1and the players perceived that this contributed to a decrease in performance. Thirty‐three percent of players also reported a decreased appetite post‐match during P1, which has potential implications for post‐match recovery.

To our knowledge, this is the first study to assess GI damage, indirect markers of gut barrier integrity and symptoms in response to a football‐specific exercise protocol in female athletes at two distinct phases of the MC. However, we are limited in sample size due to the complexities of recruiting eumenorrheic women and scheduling study visits during P1 and P4 of consecutive cycles. Ideally, we would have conducted all the P1 visits during days 1–3 of the participants' MC, as this is when resting MC‐related GI symptoms are typically reported to be greatest (Heitkemper and Jarrett 1992; Parker et al. 2022; Brown et al. 2024) However, this brings a logistical challenge. Consequently, in P1, we managed to test participants on day 1 (*n* = 1), day 2 (*n* = 5), day 3 (*n* = 1) and day 5 (*n* = 5) of their MC. We were also unable to assess GI permeability using the gold standard method of L/R urinary excretion ratio (Ogden et al. 2020) or, as has recently been validated, plasma L/R ratio (Houghton et al. 2023) which may have provided a more precise measure of GI permeability. Future studies in this area should seek to assess post‐exercise GI permeability through L/R plasma or urinary excretion ratio across the MC. To ensure consistency across study visits the present study employed a 2‐h fasting period pre‐exercise. This may limit the real‐world applicability, and future studies assessing the impact of the MC on exercise‐induced GI symptoms, which employ a typical football feeding protocol, are therefore warranted.

It is also important to caveat that we observed large between‐participant variability particularly regarding their subjective ratings of GI symptoms. The results of this study may not, therefore, be applicable to all players. However, given the high incidence and severity of GI symptoms reported by some participants, it is recommended that performance support staff screen female athletes for GI symptoms, and work with athletes to identify and manage any changes in GI symptoms across the MC, particularly around exercise. Currently, a large proportion of elite female football players engage with pharmaceutical strategies, such as ibuprofen, for MC symptom management (Read et al. 2022), which may lead to side effects such as GI damage and renal complications (Brennan et al. 2021). Future research should, therefore, aim to identify non‐pharmacological strategies to mitigate GI symptoms in female athletes, particularly around exercise in P1, which could include optimising nutrition to support GI comfort. More research is also needed to understand the mechanisms behind MC‐related fluctuations in GI symptoms, which may help with the identification and development of more targeted interventions. Additionally, there is a need for longitudinal studies characterising the impact of GI symptoms across the MC on performance within an applied sport setting.

## 5 Conclusion

To conclude, GI symptoms experienced both at rest, and during a simulated football match were greater in P1 compared with P4 of the MC. This research also highlights the importance of controlling for MC phase in studies investigating GI symptoms in female athletes. There does not appear to be an association between indirect markers of gut barrier integrity and MC phase‐related changes in GI symptoms. Thus, the mechanisms contributing to elevated GI symptoms in P1 require further investigation. Future studies should also assess potential nutritional strategies to alleviate GI symptoms at rest and during exercise in P1.

## Funding

This project was funded in part through Nottingham Trent University's Vice Chancellor studentship scheme.

## Ethics Statement

All procedures were performed in accordance with the Declaration of Helsinki and were approved by the Nottingham Trent University Human Invasive Ethics Committee (REF: 702).

## Conflicts of Interest

The authors declare no conflicts of interest.

## Data Availability

The data that support the findings of this study are available from the corresponding author upon reasonable request.

## References

[ejsc70157-bib-0001] Armour, M. , K. A. Parry , K. Steel , and C. A. Smith . 2020. “Australian Female Athlete Perceptions of the Challenges Associated With Training and Competing When Menstrual Symptoms Are Present.” International Journal of Sports Science & Coaching 15, no. 3: 316–323. 10.1177/1747954120916073.

[ejsc70157-bib-0002] Åstrand, P. , Rodahl, K. , Dahl, H. A. and Strømme, S. B. , 2003. Textbook of Work Physiology: Physiological Bases of Exercise. 4th ed. Human Kinetics.

[ejsc70157-bib-0003] Borg, G. A. 1982. “Psychophysical Bases of Perceived Exertion.” Medicine & Science in Sports & Exercise 14, no. 5: 377–381. 10.1249/00005768-198205000-00012. 7154893

[ejsc70157-bib-0004] Braniste, V. , M. Leveque , C. Buisson‐Brenac , L. Bueno , J. Fioramonti , and E. Houdeau . 2009. “Oestradiol Decreases Colonic Permeability Through Oestrogen Receptor Beta‐Mediated Up‐Regulation of Occludin and Junctional Adhesion Molecule‐A in Epithelial Cells.” Journal of Physiology 587, no. Pt 13: 3317–3328. 10.1113/jphysiol.2009.169300. 19433574 PMC2727039

[ejsc70157-bib-0005] Brennan, R. , M. Wazaify , H. Shawabkeh , I. Boardley , J. McVeigh , and M. C. Van Hout . 2021. “A Scoping Review of Non‐Medical and Extra‐Medical Use of Non‐Steroidal Anti‐Inflammatory Drugs (NSAIDs).” Drug Safety 44, no. 9: 917–928. 10.1007/s40264-021-01085-9. 34331260 PMC8370940

[ejsc70157-bib-0006] Brown, G. A. , M. Jones , B. Cole , A. Shawdon , and R. Duffield . 2024. “Self‐Reported Menstrual Health, Symptomatology, and Perceived Effects of the Menstrual Cycle for Elite Junior and Senior Football Players.” International Journal of Sports Physiology and Performance 19, no. 10: 1012–1020. 10.1123/ijspp.2023-0522. 39089677

[ejsc70157-bib-0007] Bruinvels, G. , E. Goldsmith , R. Blagrove , et al. 2021. “Prevalence and Frequency of Menstrual Cycle Symptoms Are Associated With Availability to Train and Compete: A Study of 6812 Exercising Women Recruited Using the Strava Exercise App.” British Journal of Sports Medicine 55, no. 8: 438–443. 10.1136/bjsports-2020-102792. 33199360

[ejsc70157-bib-0008] Camilleri, M. , A. Nadeau , J. Lamsam , et al. 2010. “Understanding Measurements of Intestinal Permeability in Healthy Humans With Urine Lactulose and Mannitol Excretion.” Neuro‐Gastroenterology and Motility 22, no. 1: 15. 10.1111/j.1365-2982.2009.01361.x. PMC2802677 19614866

[ejsc70157-bib-0009] Chantler, S. , A. Griffiths , J. Matu , G. Davison , B. Jones , and K. Deighton . 2021. “The Effects of Exercise on Indirect Markers of Gut Damage and Permeability: A Systematic Review and Meta‐analysis.” Sports Medicine 51, no. 1: 113–124. 10.1007/s40279-020-01348-y. 33201454 PMC7806566

[ejsc70157-bib-0010] Chantler, S. , R. Wood‐Martin , A. Holliday , et al. 2024. “The Frequency and Severity of Gastrointestinal Symptoms in Rugby Players.” International Journal of Sports Medicine 45, no. 4. 10.1055/a-2206-4751. 38272040

[ejsc70157-bib-0011] Cohen, J. W. 1988. Statistical Power Analysis for the Behavioral Sciences. 2nd ed. Lawrence Erlbaum Associates.

[ejsc70157-bib-0012] Costa, R. J. S. , R. M. J. Snipe , C. M. Kitic , and P. R. Gibson . 2017. “Systematic Review: Exercise‐induced Gastrointestinal Syndrome—Implications for Health and Intestinal Disease.” Alimentary Pharmacology & Therapeutics 46, no. 3: 246–265. 10.1111/apt.14157. 28589631

[ejsc70157-bib-0013] de Oliveira, E. P. , and R. C. Burini . 2009. “The Impact of Physical Exercise on the Gastrointestinal Tract.” Current Opinion in Clinical Nutrition and Metabolic Care 12, no. 5: 533–538. 10.1097/MCO.0b013e32832e6776. 19535976

[ejsc70157-bib-0014] de Oliveira, E. P. , R. C. Burini , and A. Jeukendrup . 2014. “Gastrointestinal Complaints During Exercise: Prevalence, Etiology, and Nutritional Recommendations.” Sports Medicine 44, no. 1: 79–85. 10.1007/s40279-014-0153-2. PMC4008808 24791919

[ejsc70157-bib-0015] Elliott‐Sale, K. J. , C. L. Minahan , X. A. K. J. de Jonge , et al. 2021. “Methodological Considerations for Studies in Sport and Exercise Science With Women as Participants: A Working Guide for Standards of Practice for Research on Women.” Sports Medicine 51, no. 5: 843–861. 10.1007/s40279-021-01435-8. 33725341 PMC8053180

[ejsc70157-bib-0016] Flood, T. R. , M. R. Kuennen , S. D. Blacker , S. D. Myers , E. F. Walker , and B. J. Lee . 2022. “The Effect of Sex, Menstrual Cycle Phase and Oral Contraceptive Use on Intestinal Permeability and Ex‐Vivo Monocyte TNFα Release Following Treatment With Lipopolysaccharide and Hyperthermia.” Cytokine 158: 155991. 10.1016/j.cyto.2022.155991. 35944412

[ejsc70157-bib-0017] Gaskell, S. K. , R. M. J. Snipe , and R. J. S. Costa . 2019. “Test‐Retest Reliability of a Modified Visual Analog Scale Assessment Tool for Determining Incidence and Severity of Gastrointestinal Symptoms in Response to Exercise Stress.” International Journal of Sport Nutrition and Exercise Metabolism 29, no. 4: 411–419. 10.1123/ijsnem.2018-0215. 30632417

[ejsc70157-bib-0018] Greig, M. P. , L. R. McNaughton , and R. J. Lovell . 2006. “Physiological and Mechanical Response to Soccer‐Specific Intermittent Activity and Steady‐State Activity.” Research in Sports Medicine 14, no. 1: 29–52. 10.1080/15438620500528257. 16700403

[ejsc70157-bib-0019] Groschwitz, K. R. , and S. P. Hogan . 2009. “Intestinal Barrier Function: Molecular Regulation and Disease Pathogenesis.” Journal of Allergy and Clinical Immunology 124, no. 1: 3–20. 10.1016/j.jaci.2009.05.038. 19560575 PMC4266989

[ejsc70157-bib-0020] Günzel, D. , and A. S. L. Yu . 2013. “Claudins and the Modulation of Tight Junction Permeability.” Physiological Reviews 93, no. 2: 525–569. 10.1152/physrev.00019.2012. 23589827 PMC3768107

[ejsc70157-bib-0021] Heitkemper, M. M. , and M. Jarrett . 1992. “Pattern of Gastrointestinal and Somatic Symptoms Across the Menstrual Cycle.” Gastroenterology 102, no. 2: 505–513. 10.1016/0016-5085(92)90097-i. 1732122

[ejsc70157-bib-0022] Houghton, M. l. J. , R. M. J. Snipe , G. Williamson , and R. J. S. Costa . 2023. “Plasma Measurements of the Dual Sugar Test Reveal Carbohydrate Immediately Alleviates Intestinal Permeability Caused by Exertional Heat Stress.” Journal of Physiology 601, no. 20: 4573–4589. 10.1113/JP284536. 37695123

[ejsc70157-bib-0023] Judkins, T. C. , J. C. Dennis‐Wall , S. M. Sims , J. Colee , and B. Langkamp‐Henken . 2020. “Stool Frequency and Form and Gastrointestinal Symptoms Differ by Day of the Menstrual Cycle in Healthy Adult Women Taking Oral Contraceptives: A Prospective Observational Study.” BMC Women's Health 20, no. 1: 136. 10.1186/s12905-020-01000-x. 32600463 PMC7325082

[ejsc70157-bib-0024] Lambert, G. P. 2009. “Stress‐Induced Gastrointestinal Barrier Dysfunction and Its Inflammatory Effects.” Supplement, Journal of Animal Science 87, no. S14: 101–108. 10.2527/jas.2008-1339. 18791134

[ejsc70157-bib-0025] Larsen, B. , K. Morris , K. Quinn , M. Osborne , and C. Minahan . 2020. “Practice Does Not Make Perfect: A Brief View of Athletes’ Knowledge on the Menstrual Cycle and Oral Contraceptives.” Journal of Science and Medicine in Sport 23, no. 8: 690–694. 10.1016/j.jsams.2020.02.003. 32089432

[ejsc70157-bib-0026] Leiper, J. B. 2015. “Fate of Ingested Fluids: Factors Affecting Gastric Emptying and Intestinal Absorption of Beverages in Humans.” Supplement, Nutrition Reviews 73, no. S2: 57–72. 10.1093/nutrit/nuv032. 26290292

[ejsc70157-bib-0027] Li, J. Y. , B. Chassaing , A. M. Tyagi , et al. 2016. “Sex Steroid Deficiency‐Associated Bone Loss is Microbiota Dependent and Prevented by Probiotics.” Journal of Clinical Investigation 126, no. 6: 2049–2063. 10.1172/JCI86062. 27111232 PMC4887186

[ejsc70157-bib-0028] Martin, D. , C. Sale , S. B. Cooper , and K. J. Elliott‐Sale . 2018. “Period Prevalence and Perceived Side Effects of Hormonal Contraceptive Use and the Menstrual Cycle in Elite Athletes.” International Journal of Sports Physiology and Performance 13, no. 7: 926–932. 10.1123/ijspp.2017-0330. 29283683

[ejsc70157-bib-0029] McKay, A. K. A. , T. Stellingwerff , E. S. Smith , et al. 2022. “Defining Training and Performance Caliber: A Participant Classification Framework.” International Journal of Sports Physiology and Performance 17, no. 2: 317–331. 10.1123/ijspp.2021-0451. 34965513

[ejsc70157-bib-0030] McKenna, Z. J. , Z. J. Fennel , Q. N. Berkemeier , et al. 2022. “Exercise in Hypobaric Hypoxia Increases Markers of Intestinal Injury and Symptoms of Gastrointestinal Distress.” Experimental Physiology 107, no. 4: 326–336. 10.1113/EP090266. 35224797

[ejsc70157-bib-0031] McKenna, Z. J. , J. Houck , J. Ducharme , et al. 2022. “The Effect of Prolonged Interval and Continuous Exercise in the Heat on Circulatory Markers of Intestinal Barrier Integrity.” European Journal of Applied Physiology 122, no. 12: 2651–2659. 10.1007/s00421-022-05049-4. 36114840

[ejsc70157-bib-0032] McNulty, K. L. , P. Ansdell , S. Goodall , et al. 2023. “The Symptoms Experienced by Naturally Menstruating Women and Oral Contraceptive Pill Users and Their Perceived Effects on Exercise Performance and Recovery Time Posttraining.” Women in Sport & Physical Activity Journal 32, no. 1: 1–13. 10.1123/wspaj.2023-0016.

[ejsc70157-bib-0033] Meleine, M. , and J. Matricon . 2014. “Gender‐Related Differences in Irritable Bowel Syndrome: Potential Mechanisms of Sex Hormones.” World Journal of Gastroenterology 20, no. 22: 6725–6743. 10.3748/wjg.v20.i22.6725. 24944465 PMC4051914

[ejsc70157-bib-0034] Micklewright, D. , A. St Clair Gibson , V. Gladwell , and A. Al Salman . 2017. “Development and Validity of the Rating‐of‐Fatigue Scale.” Sports Medicine 47, no. 11: 2375–2393. 10.1007/s40279-017-0711-5. 28283993 PMC5633636

[ejsc70157-bib-0035] Nie, X. , R. Xie , and B. Tuo . 2018. “Effects of Estrogen on the Gastrointestinal Tract.” Digestive Diseases and Sciences 63, no. 3: 583–596. 10.1007/s10620-018-4939-1. 29387989

[ejsc70157-bib-0036] Nieman, D. C. , D. A. Henson , C. L. Dumke , et al. 2006. “Ibuprofen Use, Endotoxemia, Inflammation, and Plasma Cytokines During Ultramarathon Competition.” Brain, Behavior, and Immunity 20, no. 6: 578–584. 10.1016/j.bbi.2006.02.001. 16554145

[ejsc70157-bib-0037] Ogden, H. B. , J. L. Fallowfield , R. B. Child , et al. 2020. “Reliability of Gastrointestinal Barrier Integrity and Microbial Translocation Biomarkers at Rest and Following Exertional Heat Stress.” Physiological Reports 8, no. 5: e14374. 10.14814/phy2.14374. 32170836 PMC7070100

[ejsc70157-bib-0038] Parker, L. J. , K. J. Elliott‐Sale , M. P. Hannon , J. P. Morton , and G. L. Close . 2022. “An Audit of Hormonal Contraceptive Use in Women's Super League Soccer Players; Implications on Symptomology.” Science & Medicine in Football 6, no. 2: 153–158. 10.1080/24733938.2021.1921248. 35475746

[ejsc70157-bib-0039] Parnell, J. A. , K. Wagner‐Jones , R. F. Madden , and K. A. Erdman . 2020. “Dietary Restrictions in Endurance Runners to Mitigate Exercise‐Induced Gastrointestinal Symptoms.” Journal of the International Society of Sports Nutrition 17, no. 1: 32. 10.1186/s12970-020-00361-w. 32522222 PMC7288429

[ejsc70157-bib-0040] Pelsers, M. M. A. L. , Z. Namiot , W. Kisielewski , et al. 2003. “Intestinal‐Type and Liver‐Type Fatty Acid‐Binding Protein in the Intestine: Tissue Distribution and Clinical Utility.” Clinical Biochemistry 36, no. 7: 529–535. 10.1016/S0009-9120(03)00096-1. 14563446

[ejsc70157-bib-0041] Pugh, J. N. , K. M. Lydon , O. O'Donovan , and S. M. Madigan . 2022. “More Than a Gut Feeling: What is the Role of the Gastrointestinal Tract in Female Athlete Health?” European Journal of Sport Science 22, no. 5: 755–764. 10.1080/17461391.2021.1921853. 33944684

[ejsc70157-bib-0042] Pugh, J. N. , A. S. Sparks , D. A. Doran , et al. 2019. “Four Weeks of Probiotic Supplementation Reduces GI Symptoms During a Marathon Race.” European Journal of Applied Physiology 119, no. 7: 1491–1501. 10.1007/s00421-019-04136-3. 30982100 PMC6570661

[ejsc70157-bib-0043] Read, P. , R. Mehta , C. Rosenbloom , E. Jobson , and K. Okholm Kryger . 2022. “Elite Female Football Players' Perception of the Impact of Their Menstrual Cycle Stages on Their Football Performance. A Semi‐Structured Interview‐Based Study.” Science & Medicine in Football 6, no. 5: 616–625. 10.1080/24733938.2021.2020330. 36540911

[ejsc70157-bib-0044] Smith, E. S. , R. McCormick , A. K. A. McKay , et al. 2025. “Perceived Negative Menstrual Cycle Symptoms, But Not Changes in Estrogen or Progesterone, Are Associated With Impaired Cycling Race Performance.” Medicine & Science in Sports & Exercise 57, no. 3: 590–599. 10.1249/MSS.0000000000003587. 39501484

[ejsc70157-bib-0045] Smith, K. A. , J. N. Pugh , F. A. Duca , G. L. Close , and M. J. Ormsbee . 2021. “Gastrointestinal Pathophysiology During Endurance Exercise: Endocrine, Microbiome, and Nutritional Influences.” European Journal of Applied Physiology 121, no. 10: 2657–2674. 10.1007/s00421-021-04737-x. 34131799

[ejsc70157-bib-0046] Torella, M. , N. Colacurci , P. De Franciscis , et al. 2007. “Intestinal Permeability in Healthy Women During the Menstrual Cycle and in the Postmenopause.” Italian Journal of Gynaecology and Obstetrics 19: 17–20. https://hdl.handle.net/11591/204217.

[ejsc70157-bib-0047] van der Giessen, J. , C. J. van der Woude , M. P. Peppelenbosch , and G. M. Fuhler . 2019. “A Direct Effect of Sex Hormones on Epithelial Barrier Function in Inflammatory Bowel Disease Models.” Cells 8, no. 3: 261. 10.3390/cells8030261. 30893871 PMC6468635

[ejsc70157-bib-0048] van Wijck, K. , K. Lenaerts , L. J. van Loon , W. H. Peters , W. A. Buurman , and C. H. Dejong . 2011. “Exercise‐Induced Splanchnic Hypoperfusion Results in Gut Dysfunction in Healthy Men.” PLoS One 6, no. 7: e22366. 10.1371/journal.pone.0022366. 21811592 PMC3141050

[ejsc70157-bib-0049] Whitcomb, B. W. , S. L. Mumford , N. J. Perkins , et al. 2014. “Urinary Cytokine and Chemokine Profiles Across the Menstrual Cycle in Healthy Reproductive‐Aged Women.” Fertility and Sterility 101, no. 5: 1383–1391. 10.1016/j.fertnstert.2014.01.027. 24581581 PMC4008697

[ejsc70157-bib-0050] Wilson, P. B. , R. Fearn , and J. Pugh . 2023. “Occurrence and Impacts of Gastrointestinal Symptoms in Team‐Sport Athletes: A Preliminary Survey.” Clinical Journal of Sport Medicine: Official Journal of the Canadian Academy of Sport Medicine 33, no. 3: 239–245. 10.1097/JSM.0000000000001113. 36476634

[ejsc70157-bib-0051] Wright, S. D. , R. A. Ramos , P. S. Tobias , R. J. Ulevitch , and J. C. Mathison . 1990. “CD14, a Receptor for Complexes of Lipopolysaccharide (LPS) and LPS Binding Protein.” Science 249, no. 4975: 1431–1433. 10.1126/science.1698311. 1698311

[ejsc70157-bib-0052] Yeh, Y. J. , L. Y. L. Law , and C. L. Lim . 2013. “Gastrointestinal Response and Endotoxemia During Intense Exercise in Hot and Cool Environments.” European Journal of Applied Physiology 113, no. 6: 1575–1583. 10.1007/s00421-013-2587-x. 23314685

